# Experimental device to measure the compressibility coefficient of soft materials

**DOI:** 10.1371/journal.pone.0322716

**Published:** 2025-05-29

**Authors:** N. Briot, G. Chagnon, N. Connesson, T. Alonso, P.-A. Barraud, Y. Payan

**Affiliations:** Univ. Grenoble Alpes, CNRS, UMR 5525, VetAgro Sup, Grenoble INP, TIMC, 38000 Grenoble, France; Universiti Teknologi Petronas: Universiti Teknologi PETRONAS, MALAYSIA

## Abstract

At present, there are many ways to measure the compressibility coefficient of a sample, but none of them allow the measurement of compressibility on samples that cannot be cut to a precise shape, such as human soft tissue, rubber-like materials and polymers. The objective of this study is therefore to demonstrate the proof of concept of a device allowing the measurement of the compressibility coefficient on a sample which is non-cuttable in a precise shape. The device is made of a cylindrical chamber, filled with a liquid, in which a sample of the soft material is inserted. The volume of the chamber is decreased by means of the insertion of a piston while the resulting pressure variation is measured. The compressibility coefficient of the soft material is then estimated from the pressure-volume curves. The results obtained on two industrial materials, namely a PMMA and a SBR rubber, show that the method produces similar results than those obtain by a classical stereocorrelation analysis on a tensile test. These results give confidence in the coefficients obtained with the compressibility method and open perspectives for human soft tissues.

## 1 Introduction

Many soft materials are considered as incompressible, like human soft tissues, rubber-like materials and polymers. As a consequence, their compressibility coefficients are often assumed very low. The classical way to treat these materials in theoretical developments or finite element models is to consider their Poisson’s ratio close to 0.5. There is however an important gap in the literature since few Poisson’s ratios or compressibility coefficients have really been experimentally measured. The present study aims at filling this gap with the development of an experimental device able to measure the compressibility coefficient of soft materials.

The experimental techniques used to measure compressibility coefficients or Poisson’s ratio strongly depend on the materials studied. To begin, many studies concern soils. For example, Asaei and Moosavi’s [1] developed a system for estimating the compressibility coefficient and porosity of composite mineral materials using a hydraulic injection system. To measure compressibility, the pressure is recorded as a function of the volume of liquid injected by a pump into a chamber containing the material sample.

For polymers, various methods have been proposed. As a first example, De Crevoisier *et al*. [2] performed a filmed mechanical test. A standard size sample is subjected to a mechanical test (tension or compression) while high definition cameras record the face and edge of the sample. After processing, the volume change can be measured as a function of the force applied to the sample. There are other original types of measurements, such as Gurvich and Fleichman’s [3], who performed a compression test on a cylindrical sample. Video recordings of the curvature of the sample (front view) are then combined with a numerical modelling method, in order to model this curvature and thus deduce the volume change. Similarly, Copeland [4] proposed an original method by applying a pressure variation in a chamber containing a sample and water and measuring the volume variation using valves. Gee *et al*. [5] proposed to use hydrostatic weighing to measure the variation in weight (and therefore in volume) of a standard size specimen undergoing a tensile test while immersed in water.

As concerns soft materials such as elastomers, three standard methods have been proposed to estimate the compressibility coefficient: the wave measurement method, the oedometer compression and mechanical testing in a closed environment.

As for the wave measurement method, the compressibility coefficient is deduced by analysing the time taken for a wave to propagate through an environment. Maia *et al*. [6] used this method with ultrasonic waves sent into the sample to measure the change in compressibility coefficient during the curing of a resin. With the same wave method, Li *et al*. [7] compared the compressibility of tridecane with that of a reference liquid using a vibrating tube densimeter. Similarly, Edmonds and Nolle [8] measured the compressibility of a polymer with a wave vibration sent first in a chamber containing a reference fluid only and then sent in a chamber containing the same fluid and the polymer sample.

The method for measuring the compressibility with oedometric compression consists of a compression test run in a closed environment where the sample occupies the entire space, coupled with the measurement of the volume change. Plachy *et al*. [9] and Warfield [10] used this method on rubber and polymer respectively.

The third method to estimate the compressibility coefficient of soft materials is a mechanical stress test performed on a standard size sample in a chamber filled with an incompressible fluid. The volume change during the test can therefore be measured. Zimmermann and Stommel [11], Penn [12], Jones and Yiengst [13] and Shinomura and Takahashi [14] all performed the same type of tests on various rubbers. They placed a rubber sample with a standard shape (dog bones for Zimmermann and Stommel [11], loop for the others) in a chamber filled with an incompressible fluid. Then a tensile test was performed while the volume variation was read on a capillary. Shuttleworth [15] performed the same type of experiment but on a cylindrical rubber sample. Carew *et al*. [16] were among the rare authors to perform tests on biological tissues. They used part of an artery placed in a chamber filled with an incompressible fluid. A known volume of fluid was injected into the artery while the volume change was read on a capillary.

Finally, in order to avoid the presence of a device performing a mechanical stress on the sample inside the chamber, three interesting studies [17–19] proposed an alternative method. A sample, which volume was measured before the experiment, was placed in a chamber full of a fluid of known compressibility. The volume of the chamber was then reduced and the change in pressure was measured as a function of the change in volume. These three studies are interesting, although the constraint of a small size for the sample can be problematic in the case of human soft tissue.

Most methods described above require the sample to be cut with a very precise shape before being tested. This can be problematic, first because it is not always easy to manufacture standardised specimens, and second because very small variations of the specimen geometry can have strong consequences on the values estimated for the compressibility coefficients. If we extrapolate to biological soft tissues that are targeted in this paper, the precise manufacture of standardised specimens is often impossible. Chen *et al*. [20], for their part, describe an interesting study on a device that makes it possible to dispense with the cutting of a standard-sized sample by performing an inverse compressible hyperelastic law analysis on in vivo measurements. However there is no direct measurement of the change in volume.

The present study aims at proposing a device capable of measuring the compressibility coefficient of soft materials with the application to biological soft tissues with samples that can have very complex shapes. With a sample of known volume immersed in a liquid of known compressive behaviour, this device enables the modulus of elasticity to be measured directly. The device should also provide accurate measurements in the case of compressibility coefficients that range between high values (compressible materials) and that of the water (quasi-incompressible materials). The objective of this paper is to present a proof of concept to measure the compressibility coefficient of soft materials and the resulting Poisson’s ratios. For this purpose, a double-checking protocol using two measurement methods was set up. In the first part of the paper, the experimental device is presented while in the second part of the paper, the experimental validation is detailed using two different materials. Then, a discussion is carried out.

## 2 Materials and methods

The objective of this section is to develop an experimental set-up measuring compressibility coefficients of materials that can adapt to samples with various geometrical forms.

### 2.1 Materials

Here an in-depth description of the device, as well as the tested materials, is given.

#### 2.1.1 Device description.

A compressibility test can be summarised as the determination of the relationship between pressure variation and volume variation. In most experimental studies, the system imposes a pressure variation on the sample and measures the resulting volume variation, as in the works of Copeland [4] and Asaei and Moosavi [1]. In the present study, the choice was made to impose a volume variation and to measure the resulting pressure variation. Such a choice comes from the example of the oedometer tests developed by Taylor [21] and applied to the brain by Franceschini *et al*. [22]. As soft tissues (e.g., adipose tissue) cannot be cut into perfectly cylindrical samples, the device was adapted with the use of a chamber where the sample is inserted while the chamber is filled by a liquid. An actuator is used to reduce the volume of the chamber and to impose volume variations. The control of the actuator can be transferred to a conventional tension/compression machine, here a MTS machine model C42 503.

**Fig 1:**
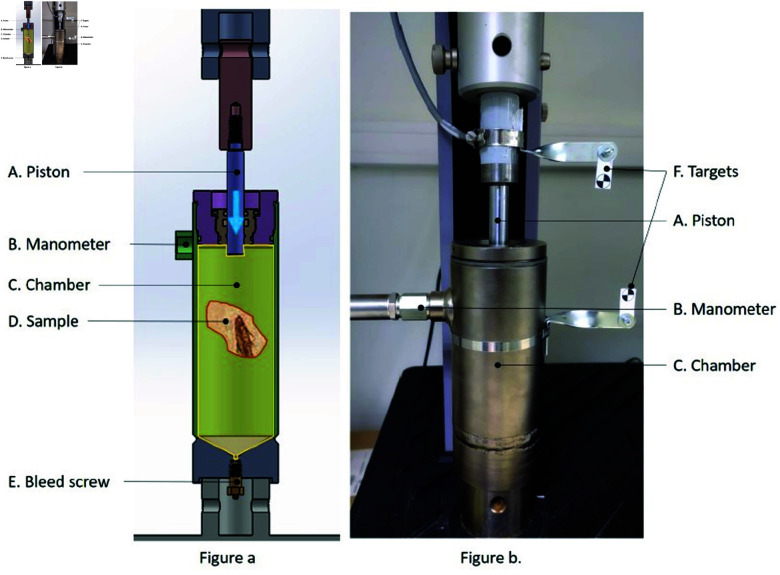
Compressibility measurement device. (**a**) Sketch of the device. (**b**) Image of the device.

In Fig 1, the device is presented with labels for each part of the system. The sample D is placed in the chamber C that is filled with water (but another liquid can be used such as a saline solution or any kind of oil). It is important that the chamber is only filled with the sample and the liquid chosen for the study, so a bleed screw E was installed to evacuate the air present in the system before starting the measurements.

Once the system is installed in the test rig, the piston A is used to reduce the volume of C. The diameter of A was reduced as much as possible in order to maximise the vertical displacement and minimise the volume variation, thus increasing measurement accuracy. The pressure gauge B allows the pressure in C to be measured directly. The whole system is based on the modification of an industrial hydraulic piston to ensure that no leakage can occur (based on lip seals and an O-ring seal), which would have a strong impact on the measurements.

#### 2.1.2 Tested materials.

Our aim is to test the device on materials with very different volume variation capabilities. Even though the device is adapted to materials with complex shapes, it was decided in a first step to test materials with regular shapes in order to be able to compare the results of the device with other more conventional means of measurement. Two materials were chosen for this test. First, a Polymethyl methacrylate (PMMA XT) material was investigated, with a Poisson’s ratio that is announced between 0.35 and 0.45 in the literature. Second, a Styrene-Butadiene Rubber (SBR) elastomer filled with carbon black material was used as a test material (Poisson’s ratio very close to 0.5 depending on the SBR [23]). Such an elastomer is indeed considered as a good approximation of some biological soft tissues that are often assumed as quasi-incompressible.

### 2.2 Methods

#### 2.2.1 Protocol description.

**Fig 2:**
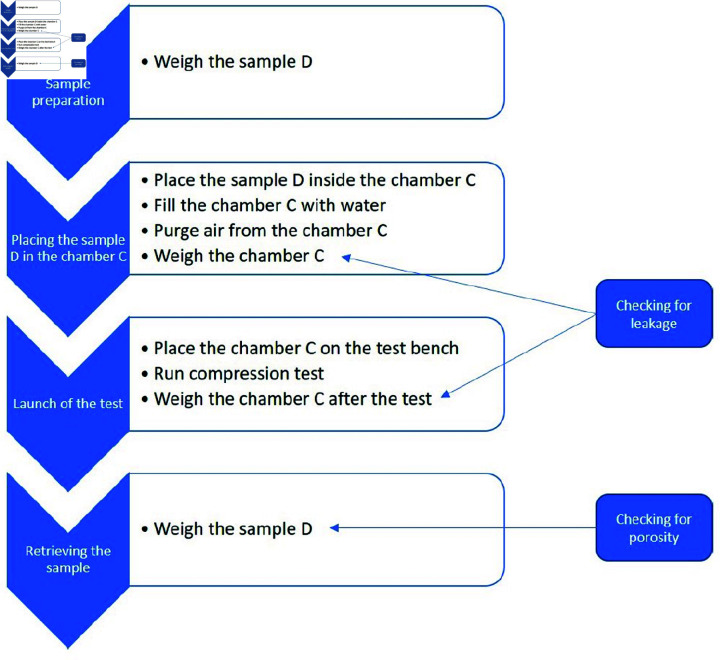
Protocol for compressibility measurement.

The developed system requires the use of a strict protocol to set up the test in order to avoid disturbances in the measurements and results. The whole process is described in Fig 2. A sample of any shape can be used, and simply needs to be weighed. The sample is placed in chamber C, which is then filled with the chosen liquid. An important step is the extraction of the maximum amount of air trapped in the chamber through a purge system (the small remaining bubbles will be dissolved in the water as the pressure increases, as corresponds to the setting in Fig 1). The whole system is then weighed and placed in a conventional tension/compression machine to perform the compressibility test. Two final steps are required; the first is to weigh the system. This reference weight will be used to ensure that the system weight is unchanged at the test end and that no leakage has occurred. The second step consists of removing the sample from the device and weighing it to ensure that no liquid absorption has occurred (ensure there is no porosity in the sample). If this is not the case, either the sample is considered invalid, or the experiment is repeated with the same sample and the result obtained is that of the compressibility of the matrix. With these two steps verified, the test is considered valid.

Knowing the weight of C empty and the weight of C filled with water only, the volume of the sample D is deduced by:

$$V_{C}=\frac{M_{C\_full}-M_{C\_empty}}{\rho_{water}}\;V_{water}=\frac{M_{global}-(M_{C\_empty}+M_{D})}{\rho_{water}}\;V_{D}=V_{C}-V_{water}$$ (1)

With $V_{C}$ being the volume of C [mL], $M_{C\_full}$ the mass of full C [g], $M_{C\_empty}$ the mass of empty C [g], $\rho_{water}$ the density of water [${\text{g.mL}}^{-1}$], $V_{water}$ the volume of water [mL], *M*_*global*_ the total mass (C+D+water) [g], *M*_*D*_ the mass of D [g] and $V_{D}$ the volume of D [mL].

As can be seen in Fig 1b, the displacement of piston A is measured by two targets, noted F, filmed by a camera: one fixed on A and the other fixed on C. Indeed, a measurement external to the MTS (traction machine) was preferred for reasons of accuracy. The pressure variation in the chamber is measured by the manometer B, Parker pressure sensors model PTD.VB 100 1B1C1 0...100 bar.

### 2.3 Settings and influence of parameters

#### 2.3.1 Calibration.

The calibration of the various parameters of the system is necessary before performing the compressibility tests. The compressibility coefficient of water is relatively well documented [24–26]. The water will therefore serve as a reference to calibrate the system. For this study, the reference temperature will be room temperature, i.e., about 20^∘^. Considering this, the compressibility coefficient of water $\xi_{water}$ is $4.591\times 10^{-4}\;{\text{MPa}}^{-1}$ [25].

**Fig 3:**
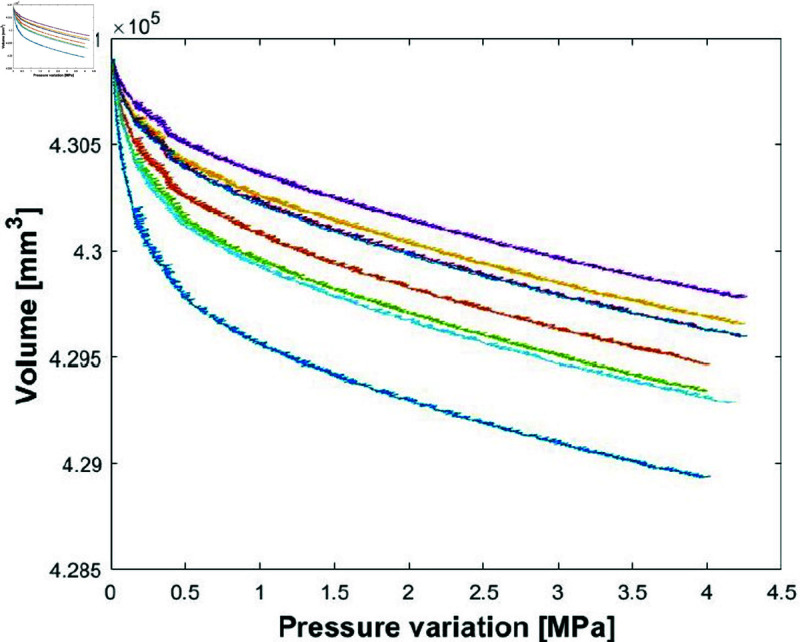
All the eight compressibility tests on water.

Eight tests were conducted with the chamber containing water only. The tests were carried out at a speed of $1.6\;\times \;10^{-3}$ %/s (percentage volume reduction per second). Fig 3 shows the corresponding pressure/volume curves obtained. The curves show a more or less pronounced curved shape for small pressure variations before having a linear shape between the volume variation and the pressure variation. The initial part corresponds to the setting up of the system and therefore its pressurisation, considering the displacement of the seals and absorption of the small remaining bubbles of gas. The initial part of the curve is therefore ignored. Only the nearly linear part, after the system has been set up, is considered for the measurement. It can be seen that all the curves are well parallel. The initial part differs from one test to the other one because the setting up of the system is complex and not replicable. The theoretical expression for the compressibility coefficient $\chi_{T}$ is given by:

$$\chi_{T}=-\frac{1}{V}{\left(\frac{\partial V}{\partial P}\right)}_{T}$$ (2)

where $\chi_{T}$ is the compressibility coefficient [${\text{MPa}}^{-1}$], *V* the volume of the sample tested [${\text{mm}}^{3}$] and *T* the temperature.

**Fig 4:**
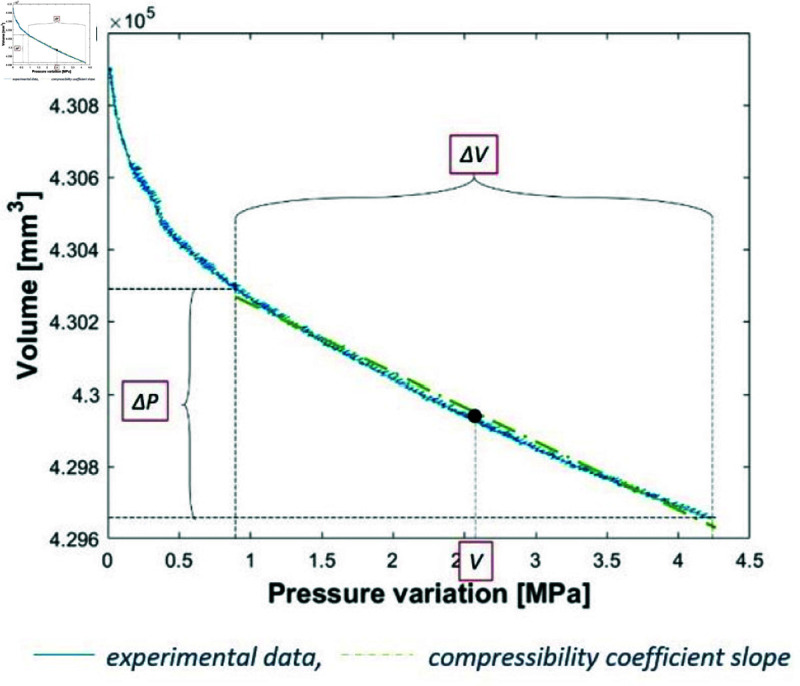
Compressibility coefficient slope measurement method.

To find the experimental compressibility coefficient for each test, a measurement point is placed in the middle of the linear part of the pressure–volume curve (Fig 4). The slope of the corresponding curve is measured assuming a linear shape for the second part (starting between 0.5 and 1 MPa depending on the test) of the curve using a least squares linear regression. This value is then divided by the middle volume between those two boundaries (Fig 4).

In the proposed system, the pressure-volume variations are due to both the volume change of the filler material and the deformation of the chamber walls and seal movements:

$$\Delta V_{imposed}=\Delta V_{content}+\Delta V_{chamber}$$ (3)

where $\Delta V_{imposed}$ is the volume variation imposed by the piston [${\text{mm}}^{3}$], $\Delta V_{content}$ the sought volume variation of the sample and filling liquid [${\text{mm}}^{3}$] and $\Delta V_{chamber}$ the volume variation of the chamber [${\text{mm}}^{3}$].

No irreversible plastic deformation of the measuring system was observed during the tests, so the chamber and seal deformations were assumed linear with the pressure loading:

$$\Delta V_{chamber}=R.\Delta P$$ (4)

Combining the last two equations, the relation simply becomes:

$$\Delta V_{content}=\Delta V_{imposed}-R.\Delta P$$ (5)

The chamber equivalent stiffness *R* is calibrated so as to obtain the correct value of the compressibility coefficient of water. The equivalent stiffness *R* is chosen to minimise the difference between the reference value and the average of the values obtained from the 8 tests. The raw compressibility value measured was $8.104\times 10^{-4}\pm 1.56\times 10^{-5}\;{\text{MPa}}^{-1}$. A linear regression was used to obtain the value of $R=141.97\;{\text{mm}}^{3}\;{\text{MPa}}^{-1}$ (Fig 4). In the end, the experimental measurement results in a compressibility coefficient for water at the desired value but with an uncertainty of $1.29\;\times \;10^{-5}{\text{MPa}}^{-1}$. This coefficient allows for the absorption of the expansion of the chamber and the displacement of the seal during pressurisation. After verification, this coefficient is fully compatible with the theoretical maximum expansion of the chamber.

#### 2.3.2 Speed influence.

The tests are intended to be carried out at low speeds to avoid any temperature rise. However, the influence of speed was evaluated, even in a low speed range. Eight tests were therefore performed with water only at a speed of $1.6\times 10^{-3}$ %/s and 5 other tests at a speed of $3.9\times 10^{-4}$ %/s. The compressibility coefficients were measured and the values are provided in Table 1.

**Table 1:** Synthesis of tests performed at $1.6\times 10^{-3}$ %/s and $3.9\times 10^{-4}$ %/s

	Means of the water compressibility coefficient [${\text{MPa}}^{-1}$]	Standard deviations [${\text{MPa}}^{-1}$]
Speed of $1.6\times 10^{-3}$ %/s	$4.591\times 10^{-4}$	$1.29\times 10^{-5}$
Speed of $3.9\times 10^{-4}$ %/s	$4.204\times 10^{-4}$	$2.19\times 10^{-5}$

A difference of 8.4% is observed between the two compression speeds, which means that speed is an important parameter to consider. A temperature measurement was made during the test, by means of a thermometer cell fixed to the outer surface of the chamber, and no major temperature variation was observed (<1^∘^C of variation). The highest compression speed ($1.6\;\times \;10^{-3}$ %/s) was chosen for all the results presented below. There will be a slight deviation in the results compared to those that would have been obtained with the lower speed, but the objective of the article is mainly to show the feasibility and the efficiency of the method. A test of the speed limit could then be carried out at a later stage.

#### 2.3.3 Other influences.

Given the development of the device, a number of precautions must be taken to ensure the quality of the measurements and to limit experimental errors.

- Influence of air inside C (Fig 1)

The presence of air bubbles in C can have a huge impact on the results due to the high compressibility coefficient of gas. This is why particular attention is given to this matter. The filling of C is done in a very delicate way by means of a long cannula. D is first wet in water and then gently placed in C. Finally, after closing C, the remaining air in C is purged through E. This solution can still be improved, but it allows the presence of air bubbles in C to be kept to a minimum. Furthermore, the presence of a small amount of air bubbles is not a severe handicap for the measurement because the bubbles are dissolved in the water during the first non-linear phase of compression (<1 MPa). There is, therefore, no impact on the measurement of the linear section of the pressure/volume slope.

- Porosity

To ensure that water does not seep into D, two weighings of D are carried out: a first weighing before the test and a second weighing after it. If the two weights are similar (±2%), it is considered that no water has penetrated D (Fig 2).

## 3 Experimental validation of the compressibility test

Compressibility tests are carried out on both test materials, i.e., PMMA and SBR. One way of validating the measurements is to identify the Poisson’s ratios of the materials from their compressibility coefficients estimated with our device and to compare them with measurements obtained in a more conventional way. In this case, a tensile test coupled with image stereo-correlation is used.

### 3.1 Compressibility tests

**Fig 5:**
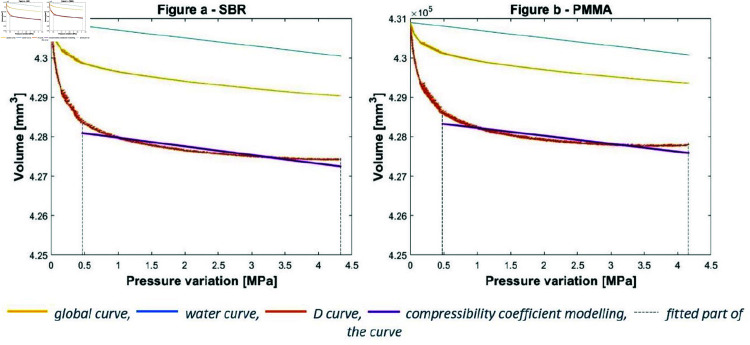
Compressibility test. (**a**) On SBR. (**b**) on PMMA.

A sample of the materials (either PMMA or SBR) is introduced into chamber C that is filled with water. The compressibility test is then performed on the material/water mixture. The curves obtained are plotted for both material/water mixtures in Fig 5a for PMMA and Fig 5b for SBR. The results should be processed to obtain the material response only, without the influence of the water. This is done by subtracting the water pressure/volume response from the global curves (corresponding to the difference in weight between a chamber filled with water and one filled with water and the material sample). For this, it is necessary to know the distribution between water and the inserted material. The volume variations $\Delta V_{water}$ and $\Delta V_{D}$ of each part can be identified as follows:

$$s=\frac{\sum_{n=1}^{\infty }p_{\chi exp\_n}}{n}\;\Delta V_{water}=s.\Delta P+V_{0}\;\Delta V_{D}=\frac{\Delta V_{global}-\Delta V_{water}.{\%}_{water}}{{\%}_{D}}$$ (6)

With *s* as the average of the pressure/volume slopes obtained for the *n* water tests $p_{\chi exp\_n}$ [${\text{mm}}^{3}.{\text{MPa}}^{-1}$], $\Delta V_{water}$ the volume variation of water, ΔP the pressure variation in C [MPa], $\Delta V_{0}$ the initial volume of D, $\Delta V_{D}$ the volume variation of D, $\Delta V_{global}$ the global volume variation, ${\%}_{water}$ the volume percentage of water in C and ${\%}_{D}$ the volume percentage of D in C. The experimental curves are processed using the formulae outlined above to remove the contribution of the water. The corresponding results are shown in Fig 5a for PMMA and Fig 5b for SBR. The observations are the same as for water in the calibration phase: the resulting curve for the sample has a very steep initial shape corresponding to the set-up of the compressive device. The second part of the curves have a more linear shape corresponding to the compression of the sample. It is this part that is used to estimate the sample compressibility coefficient (Fig 5).

### 3.2 Determination of the Poisson’s ratio of the materials

#### 3.2.1 From the compressibility measurements.

The determination of the Poisson’s ratio $\upsilon_{comp}$ from the compressibility coefficient requires the knowledge of the Young’s modulus *E* of the materials assuming a linear behaviour of the material at small strains:

$$\upsilon_{comp}=\frac{3-E.\chi_{T}}{6}$$ (7)

**Fig 6:**
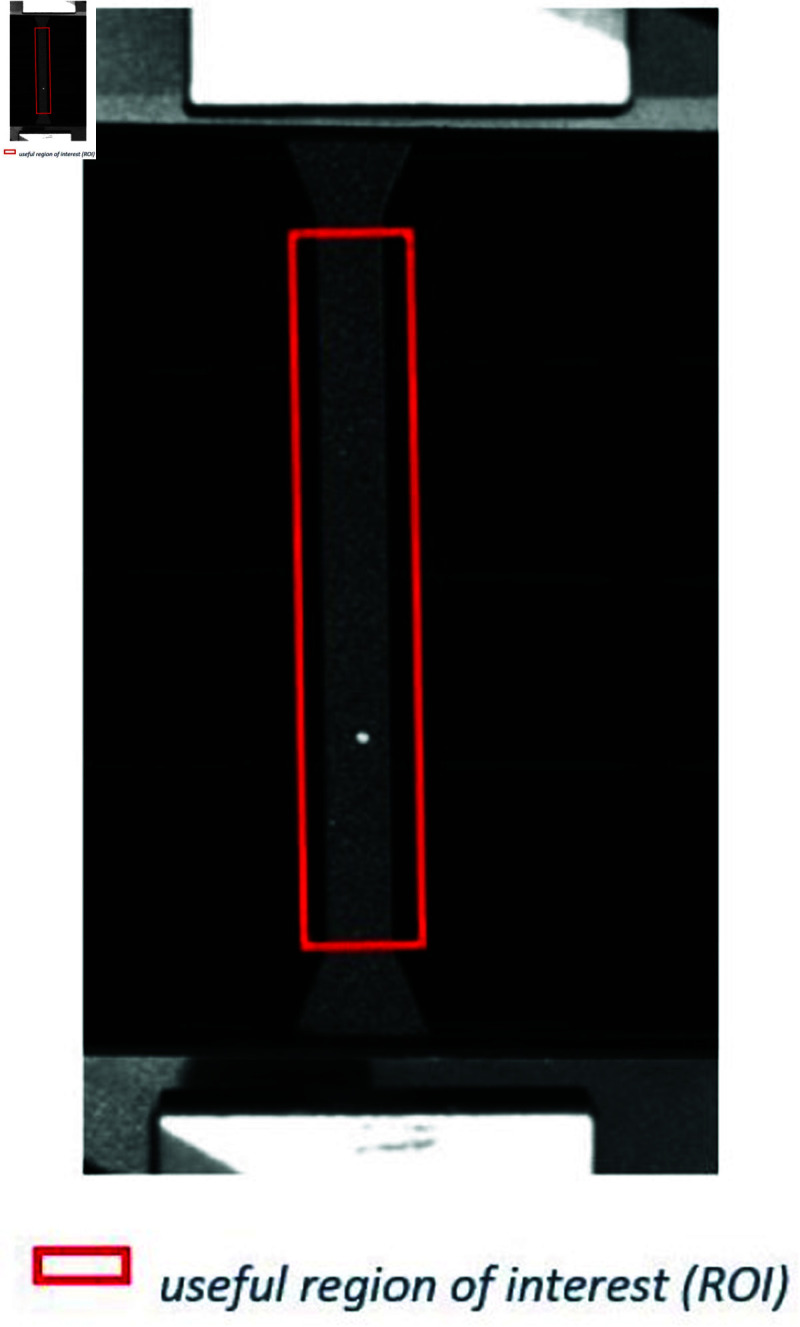
Uniaxial tensile test on PMMA samples with speckles.

Uniaxial tensile tests were performed to evaluate the specimen’s Young’s modulus. Dog-bone specimens of the materials were cut while speckles were painted on the surface of the specimens to measure their strain fields by means of a camera (digital image correlation). An illustration of a PMMA specimen with speckles on it is shown in Fig 6. The forces are measured by a 500 N load cell mounted on the MTS traction machine. The same test was repeated 10 times with a new sample to ensure accuracy.

By means of the force measurement, the nominal stress, i.e., first Piola-Kirchhoff stress, is calculated as follows:

$$\pi =\frac{F}{A_{0}}$$ (8)

where *F* is the force applied to the sample and *A*_0_ the initial cross-section area of the sample. The stretch λ is then defined by:

$$\lambda =\frac{l}{L_{0}}$$ (9)

where *l* is the current useful Region Of Interest (ROI) length of the sample and *L*_0_ is the initial useful ROI length of the sample. The nominal strain is defined as:

ε=λ−1 (10)

**Fig 7:**
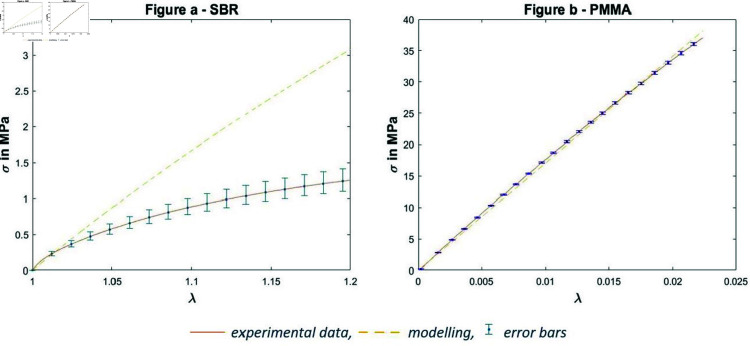
Uniaxial tensile test -a- on SBR samples -b- on PMMA

Since PMMA is stretched to small strains, the Young’s modulus can be obtained as the slope of the stress-strain curve, regardless of the choice of definition: in this case between the nominal stress and strain. The Young’s modulus *E* is identified as the slope of the corresponding lines (Fig 7b).

For the SBR samples, the curves have a non-linear shape characteristic of hyperelastic behaviour, it is therefore more complicated to identify the slope at the origin. It was decided to use a hyperelastic model to obtain the value of the Young’s modulus. The appropriate model for doing this is the neo-Hokean model [27] for which strain energy density function *W*_*Neo*_ is:

$$W_{Neo}=C_{1}(I_{1}-3)$$ (11)

where *C*_1_ is a material parameter and *I*_1_ represents the first invariant of the right Cauchy Green strain tensor. The coefficient *C*_1_ of the Neo-Hookean model is then directly related to the Young’s modulus of the material by: *C*_1_ = 6.*E* (assuming SBR as a quasi-incompressible material). As the objective was to obtain the value of the Young’s modulus, the identification of the parameter was carried out at small deformations (1<λ<1.05), the result of which is presented in Fig 7a.

#### 3.2.2 From stereo-correlation analysis.

The tensile tests used to determine the SBR and PMMA Young’s modulus were also used to directly determine optically the Poisson’s ratios of both materials. The speckle deposited on the surface of the specimens allows the measurement of all components of the strain tensor through digital image correlation. Vic Gauge 2D and VIC Snap image correlation software from ‘Kilonewton’ company were used for these measurements and the corresponding analysis. A rectangle of dimensions (*L*_0_, *l*_0_) is identified in the initial configuration; its dimensions (*L*, *l*) after deformation are identified by means of the Vic2D software; the Poisson’s ratio $\upsilon_{stereo}$ is thus simply obtained by:

$$\upsilon_{stereo}=\frac{1-\frac{l}{l_{0}}}{\frac{L}{L_{0}}-1}$$ (12)

## 4 Results

The results obtained from the two methods for the SBR and PMMA test materials are presented for comparison in Table 2. The order of magnitude of the uncertainty for the measurement of the compressibility coefficient is 3 to 4%. To our knowledge, there is no value in the literature to compare and discuss these compressibility coefficients estimated with our device. These values are then used to determine the Poisson’s ratios in combination with the Young’s modulus measured in the tensile tests (Equation (7)), whose values are also presented in the table. The obtained Poisson’s ratios are 0.376 and 0.499 for PMMA and SBR respectively. The ratios obtained by the tensile test with the image stereo-correlation method (Equation (12)) are also presented in the table: 0.363 for the PMMA and 0.480 for the SBR. The values measured by both methods are very close even though the uncertainty intervals are not intersecting. Nevertheless, the results of the compressibility measurement are fully consistent and are considered satisfactory.

**Table 2:** All the results obtained on the two test materials

		PMMA	SBR
**Compressibility method**	Volume percentage of sample in the chamber	31.3%	38.6%
	$\chi_{T}$ [${\text{MPa}}^{-1}$]	$4.32\times 10^{-4}\pm 0.30\times 10^{-4}$	$4.45\times 10^{-4}\pm 0.30\times 10^{-4}$
	$\kappa =\frac{1}{\chi_{T}}$ [MPa]	$2.31\times 10^{3}\pm 0.16\times 10^{3}$	$2.25\times 10^{3}\pm 0.15\times 10^{3}$
	Young’s Modulus *E* [MPa]	1719±26	13.71±0.34
	Poisson’s ratio $\upsilon_{comp}$	0.376±0.010	0.499±0.0001
**Stereo-correlation method**	Poisson’s ratio $\upsilon_{stereo}$	0.363±0.016	0.480±0.011

## 5 Discussion

This paper introduced an original experimental device to measure the compressibility coefficient of soft materials. The device was evaluated using water and was tested with two materials (SBR and PMMA) whose compressibility were estimated using another conventional tensile test coupled with digital image correlation. While it is original and promising, it should be emphasised that the device has nevertheless certain limitations. On one hand, the measurement depends on the ratio between the amount of sample and the amount of water in the chamber. One of the difficulties, which will need to be dealt with when moving onto human soft tissue, is to get a sample of material with a significant size inside the chamber. Indeed, in order to get as much signal as possible between the reference test (containing only water) and the test with samples and water, it is necessary to have as much sample as possible in the chamber without the sample blocking the descent of the piston A. On the other hand, the main advantage of the device is that no specific geometrical shape is required for the sample. This means that the device should be easily adapted to any material geometry, which will be a great advantage when working with specimen extracted from human soft tissues. Moreover, as previously mentioned, it will also be detrimental to the test to have the presence of air bubbles inside the sample itself; special care will have to be taken in that case to remove residual air bubbles from the system. Finally, as seen in the previous paragraphs, the load speed affects the results. Therefore, a further convergence study is required to estimate the compressibility coefficient when the load speed is infinitely small.

However, the high level of reproducibility of the test, as well as the results obtained for the SBR and PMMA materials, allows a certain amount of confidence to be placed in the use of this system for measuring a material’s compressibility coefficient. The device provides a fast and reproducible measurement using a conventional traction/compression machine with samples that do not need to have a specific shape.
